# *Vibrio cholerae* Classical Biotype Is Converted to the Viable Non-Culturable State when Cultured with the El Tor Biotype

**DOI:** 10.1371/journal.pone.0053504

**Published:** 2013-01-09

**Authors:** Subhra Pradhan, Sanjaya K. Mallick, Rukhsana Chowdhury

**Affiliations:** 1 Infectious Diseases and Immunology Division, Indian Institute of Chemical Biology, Council of Scientific and Industrial Research, Kolkata, India; 2 CU-BD Center of Excellence for Nanobiotechnology, Center for Research in Nanoscience and Nanotechnology, University of Calcutta, Kolkata, India; University of Hyderabad, India

## Abstract

A unique event in bacterial epidemiology was the emergence of the El Tor biotype of *Vibrio cholerae* O1 and the subsequent rapid displacement of the existing classical biotype as the predominant cause of epidemic cholera. We demonstrate that when the El Tor and classical biotypes were cocultured in standard laboratory medium a precipitous decline in colony forming units (CFU) of the classical biotype occurred in a contact dependent manner. Several lines of evidence including DNA release, microscopy and flow cytometric analysis indicated that the drastic reduction in CFU of the classical biotype in cocultures was not accompanied by lysis, although when the classical biotype was grown individually in monocultures, lysis of the cells occurred concomitant with decrease in CFU starting from late stationary phase. Furthermore, uptake of a membrane potential sensitive dye and protection of genomic DNA from extracellular DNase strongly suggested that the classical biotype cells in cocultures retained viability in spite of loss of culturability. These results suggest that coculturing the classical biotype with the El Tor biotype protects the former from lysis allowing the cells to remain viable in spite of the loss of culturability. The stationary phase sigma factor RpoS may have a role in the loss of culturability of the classical biotype in cocultures. Although competitive exclusion of closely related strains has been reported for several bacterial species, conversion of the target bacterial population to the viable non-culturable state has not been demonstrated previously and may have important implications in the evolution of bacterial strains.

## Introduction

From ancient civilizations to the recent Haiti epidemic [1], cholera continues to remain a public health concern particularly in developing countries where a large fraction of the population may not have access to safe drinking water and adequate sanitation. Although there are more than 200 serogroups of *Vibrio cholerae*, all the seven recorded pandemics of cholera have been caused by strains of the O1 serogroup [2], [3], [4]. The O1 serogroup can be classified into two biotypes, classical and El Tor. Strains of the classical biotype, that had probably been responsible for most of the cholera pandemics between 1817 and 1961, were in general extremely virulent and caused devastating epidemics. In 1961, the El Tor biotype emerged in Indonesia and within ten years replaced the classical biotype as the predominant cause of epidemic cholera. Indeed, the serogroup, O139 that emerged in the Indian subcontinent in 1992 to cause serious cholera epidemics has now been convincingly demonstrated to have originated from O1 El Tor [5], [6]. Although the classical and El Tor biotypes are closely related, several biochemical and genetic differences have been reported between the two biotypes. These include striking differences in carbohydrate metabolism [7], regulation of virulence gene expression [8], [9], [10], [11], virulence gene content [12], primary sequence of virulence genes [13] and susceptibility to lytic bacteriophages [14], [15], [16]. Genomic comparisons have revealed that at least 22 genes are missing in the classical biotype compared to seventh pandemic El Tor strains [12] and 524 genes have been reported to be differentially expressed between the two biotypes under conditions that induce virulence expression in the classical biotype [10]. However, in spite of the accumulation of substantial data on the differences between the classical and El Tor biotypes, the molecular and genetic mechanisms responsible for the competitive exclusion of the classical biotype following the emergence of the El Tor biotype are unknown.

Different offensive strategies are known to be adopted by bacteria for competitive elimination of related species or even subpopulations of the same species. Generally bacteriocins and other toxins secreted in the extracellular environment are used by bacteria for interspecies competition but interestingly intraspecies competitive exclusion is seen in some bacteria [17], [18]. In *E. coli*, evolved antagonistic interactions have been described that do not involve secreted toxins. Specific mutations in the *rpoS* gene encoding the stationary phase specific sigma factor have been shown to confer a growth advantage in the stationary phase (GASP) that resulted in competitive exclusion of the parental strain [19], [20]. More recently, evolution of strains with mutations in the *glgC* gene of the glycogen synthesis pathway, has been reported during serial passage of *E. coli* K-12 that can kill or inhibit the growth of ancestral cells in a process termed stationary phase contact dependent inhibition (SCDI) [21]. Although both GASP and SCDI occurred in the stationary phase, contact dependent inhibition (CDI) has also been described in *E. coli* strains in the logarithmic phase of growth [22]. Some non O1 *V. cholerae* strains possesses type VI secretion system (T6SS) and display antimicrobial properties when cocultured with several gram negative bacterial species [23], [24]. However, T6SS has been reported to be absent from the pandemic O1 serogroup.

In this study we report that when the closely related classical and El Tor biotypes of *V. cholerae* are cocultured in standard LB medium, a rapid loss of culturability of the classical biotype was observed without a significant loss of viability. Although many bacterial species including important pathogens have been shown to enter the viable non-culturable state [25], to the best of our knowledge this is the first report of conversion of a bacterial strain to the VBNC state by coculturing with a closely related strain.

## Materials and Methods

### Strains and Culture Conditions

The *V. cholerae* strains and plasmids used in this study are listed in Table S1. O395 Sm^r^ Nal^r^ was cocultured with N16961 Sm^r^, C6709 Sm^r^, E7946 and SG-24 Sm^r^ as described previously [26]. To examine if the antibiotic resistance markers affected fitness the marker was switched between the strains and O395 Sm^r^ was cocultured with N16961 Sm^r^ Nal^r^. No effect of the Nal^r^ marker on the bacterial fitness was observed.

### Quantitation of DNA in Culture Supernatants

The classical O395 and El Tor N16961 Δ*xds* Δ*dns* strains were cocultured and DNA released into culture supernatants was estimated by qPCR (details in supplementary information).

### Flow Cytometric Analysis

O395/pEGFP and El Tor N16961 were grown separately or in cocultures and GFP production was induced in O395/pEGFP cells by addition of 1 mM IPTG in the logarithmic phase of growth (O.D. 0.3). Cells were washed in phosphate buffered saline (PBS), vortexed vigorously to disrupt cellular aggregates and diluted to approx 10^6^ cells/ml. Flow cytometric analysis and sorting were performed using a BD Influx system (details in supplementary information). The sorted GFP labeled O395 cells from monocultures and cocultures were plated on LB agar and CFU per particle sorted from each gated population was determined in triplicate.

For the membrane polarization assays, the sorted cells from the monocultures and cocultures were treated with the membrane potential indicator dye JC1 (Sigma) (10 µg/ml) for 15 min and analyzed in the flow cytometer through bandpass filters 530/40 and 580/30. In some experiments the JC1 stained cells were treated with 100 µM CCCP (Sigma) for 20 min before flow cytometry.

#### Tn mutagenesis of strain O395

A transposon mutant library of classical strain O395 was constructed using plasmid pFD1 [27] (details in supplementary information). The O395 mutant pool was cocultured with the El Tor strain N16961 for 24 hours and O395 mutants (Sm^r^ Km^r^) that survived in the cocultures was selected.

## Results

### Competitive Exclusion of *V. cholerae* Classical Biotype in Cocultures with the El Tor Biotype

**Figure 1:**
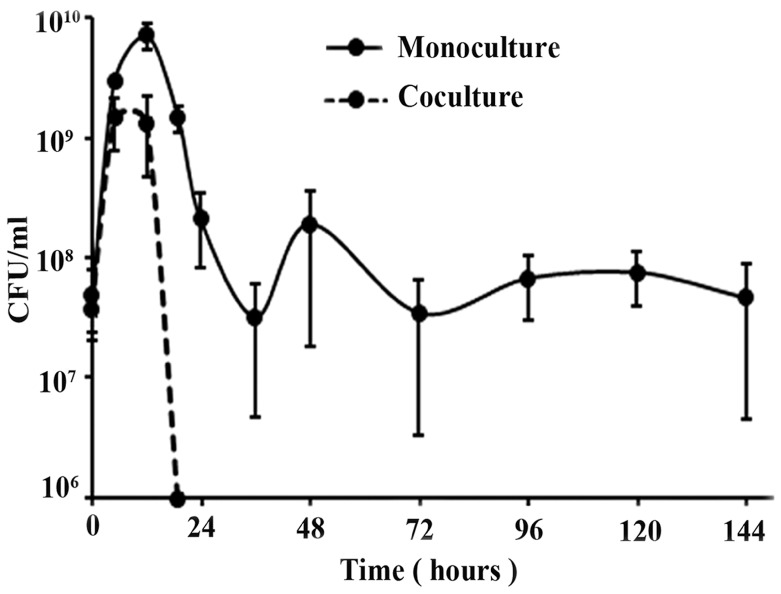
Growth of *V. cholerae* classical O395 in monocultures and cocultures. The strain O395 was grown individually in monocultures or cocultured with El Tor N16961 (1∶1) and CFU of O395 was determined at regular intervals. Data is represented as means ± SD, n = 4.

To examine the effect of coculturing the El Tor and classical biotypes of *V. cholerae*, representative strains El Tor N16961 and classical O395 were grown individually or cocultured in standard LB medium and CFU of each strain in the individual cultures and cocultures were assayed at regular intervals. As has been reported earlier [26], the El Tor N16961 strain had a growth advantage in the cocultures (data not shown). CFU of the classical strain O395 in the individual cultures and cocultures were comparable until the onset of the stationary phase when the CFU of O395 in the cocultures started decreasing more rapidly than that in the individual cultures and by 18 to 20 hours the CFU of O395 in the cocultures was more than 1000 fold lower than that in individual cultures (Fig. 1). Eventually within 48 hours, practically no O395 could be detected in the cocultures by CFU assay, although CFU of O395 in the individual culture remained at about 10^7^ to 10^8^ per ml during the 6 days examined (Fig. 1). Similar to strain N16961, the El Tor strains C6709 and E7946 and the serogroup O139 strain SG-24 (El Tor derivative) [5] were also able to outcompete the classical O395 strain in cocultures within 30 to 40 hours of mixing (Fig. S1). Furthermore, another classical strain 569B, was eliminated when cocultured with the El Tor strain N16961 (data not shown). These results suggested that the loss of CFU of the classical biotype when cocultured with the El Tor biotype may not be strain specific. All subsequent experiments were performed with the El Tor strain N16961 and the classical strain O395.

**Figure 2:**
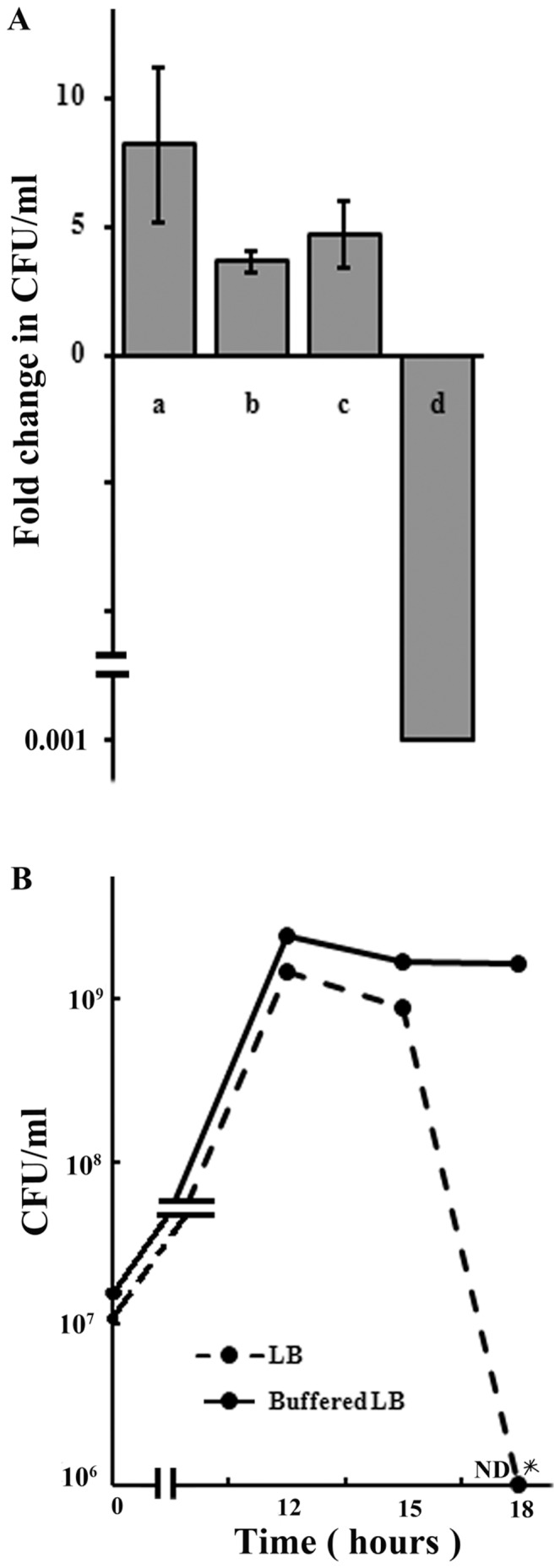
Reduction of CFU of classical biotype in cocultures is dependent on growth phase and pH. **A.** Fold change in CFU of O395 at 9 h after mixing of (a) cultures of O395 and N16961 in logarithmic phase of growth, (b) logarithmic phase culture of O395 and late stationary phase culture (24 h) of N16961 (c) late stationary phase culture of O395 and logarithmic phase culture of N16961 and (d) late stationary phase cultures of O395 and N16961. Data is represented as means ± SD, n = 3. **B.** Classical O395 and El Tor N16961 were inoculated into standard LB medium or LB buffered to pH 7 and CFU of O395 was assayed at regular intervals. The experiment was repeated twice (ND <10^4^ CFU/ml).

To determine if the observed elimination of classical O395 from the cocultures was dependent on growth phase of the cultures, O395 cells were grown to the logarithmic phase and then mixed in roughly equal proportions with the El Tor N16961 strain grown separately either to the logarithmic phase or to the stationary phase, and CFU of O395 in the cocultures was assayed at regular intervals. Also, O395 was grown to the stationary phase and mixed with log phase- or stationary phase- grown N16961 cultures. The rapid decline in CFU of O395 in the cocultures occurred only when both O395 and N16961 were in the late stationary phase (Fig. 2A). Indeed when O395 and N16961 were grown separately for 20 to 24 hours and then mixed in approximately equal proportions, greater than 1000 fold reduction in CFU of the classical O395 strain was observed within 9 hours (Fig. 2A).

In stationary phase bacterial cultures, pH of the medium is strongly alkaline due to the metabolism of amino acids as carbon source [20], [28]. Hence, the role of pH in the exclusion of the classical biotype when cocultured with the El Tor biotype was next examined. For this purpose, the two biotypes were grown together in LB medium buffered to pH 7 with HEPES (100 mM) and CFU of the classical strain O395 was monitored and compared to that in unbuffered LB medium. In unbuffered LB, by 18 hours of growth of the coculture, the pH of the medium increased to about 9 and CFU of O395 decreased to below the detectable limit (Fig. 2B). However when the two biotypes were cocultured in buffered LB medium for 18 hours, the pH of the medium was approximately 7.6 and no significant decrease in CFU of O395 was observed (Fig. 2B). It may be noted that under alkaline conditions of the stationary phase, O395 retained culturability in monocultures (Fig. 1).

These results indicated that the decline in CFU of the classical biotype when cocultured with the El Tor biotype occurred in the late stationary phase of growth under alkaline pH conditions. In all subsequent experiments, classical strain O395 and El Tor strain N16961 were grown separately for 24 hours in unbuffered LB medium, mixed in the ratio of approximately 1∶1, and cocultured for different periods of time.

### Exclusion of the Classical Biotype in Coculture is a Contact Dependent Phenomenon

When classical O395 cells were suspended in cell-free filtrates of late stationary phase cultures of El Tor N16961 or conditioned medium prepared from 24 hour cocultures of classical O395 and N16961, no significant decrease in CFU of O395 was observed (data not shown) suggesting that a secreted bacteriocin like substance was not responsible for the inhibition of the classical biotype in cocultures with the El Tor biotype.

To examine if cell-cell contact is necessary for the observed decline in CFU of O395 in cocultures with N16961, a modified version of the classical ‘U-tube’ [21] was utilized where the O395 and N16961 cultures were separated by a 0.22 µm filter unit that allowed mixing of supernatants but prohibited free mixing of the cells. The rapid decline in CFU of strain O395 was not observed under this condition (Fig. S2) suggesting that contact between classical and El Tor biotype cells was necessary for the decline in CFU of the classical biotype observed in the cocultures.

### The Decrease in CFU of the Classical Biotype in Coculture is Not Accompanied by Lysis

**Figure 3:**
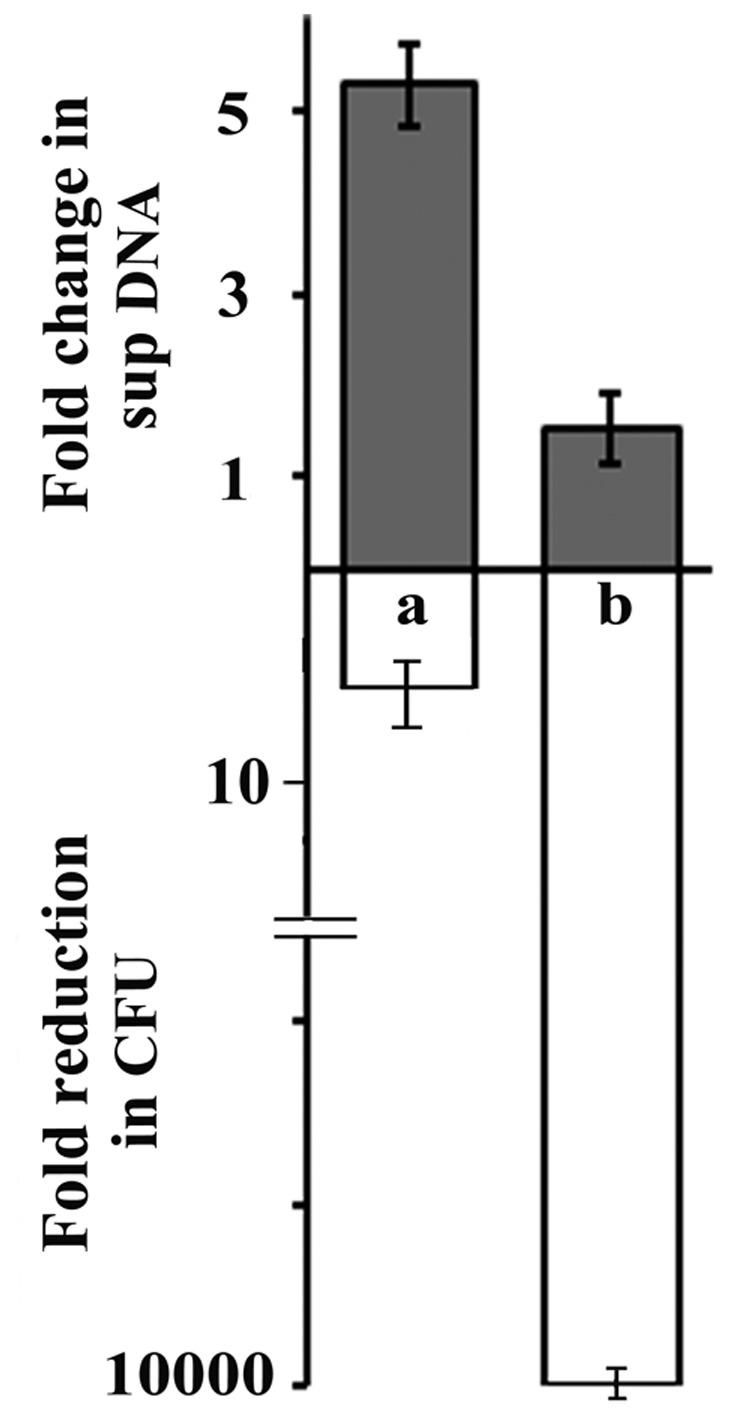
Lysis of classical O395 when grown individually in monocultures or in cocultures with El Tor N16961 Δ*dns*Δ*xds* (DNase^−^). CFU of O395 and O395 DNA in the supernatant (sup) were estimated in monocultures (a) and cocultures (b). DNA released into the supernatant when CFU of O395 in individual cultures decreased approximately six fold and that in cocultures decreased >10000 fold is indicated. Data is represented as means ± SD, n = 3.

To examine if the rapid decrease in CFU of the classical O395 strain in cocultures with the El Tor N16961 strain was due to lysis, release of O395 DNA into the supernatants of the cocultures was assayed and compared to that in individual O395 cultures. Since the El Tor N16961 secretes extracellular DNases [29] that would degrade released DNA, in this experiment a mutant strain N16961*Δdns* *Δxds* (Supplementary Information), was used that does not produce any extracellular DNase (Fig. S3). O395 and N16961Δ*dns*Δ*xds* grown separately for 24 hours were mixed, and O395 DNA released in the supernatant was assayed by quantitative real time PCR using O395 specific primers (designed from the *rstR* gene, [30], Table S2) at the beginning of and 24 hours after mixing. During this time, though CFU of O395 decreased drastically and was practically undetectable, surprisingly little increase (approximately 1.5 fold) was observed in the amount of O395 DNA released into the medium (Fig. 3). However, in individual O395 cultures, the decrease in CFU was only about 6 fold, but more than 5.5 fold increase in the amount of DNA released in the medium was observed (Fig. 3).

**Figure 4:**
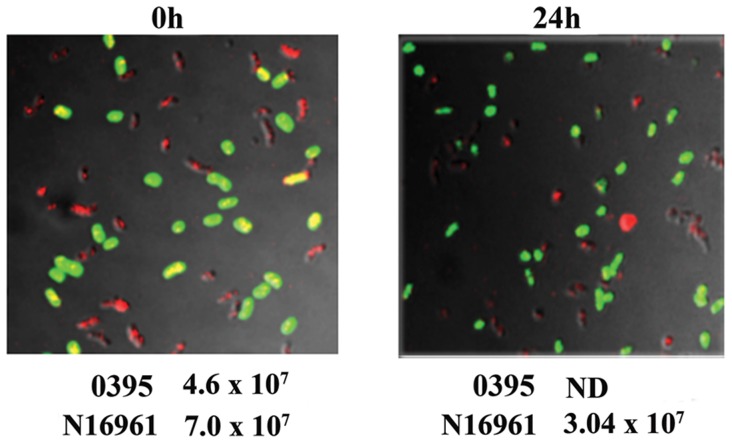
Relative proportion of classical O395 and El Tor N16961 in cocultures. GFP labeled O395 and DsRed labeled N16961 were cocultured and relative proportion of the two biotypes was determined by confocal microscopy at the time of mixing (0 h) and 24 hour after mixing. CFU of O395 and N16961 at 0 h and 24 h is indicated (ND <10^4^ CFU/ml).

In another experiment, N16961/pDsRed and O395/pEGFP (Table S1) grown separately for 24 hours were mixed in equal proportions and incubated for a further 24 hours, samples were removed for CFU assay and processed for microscopy. At the beginning of the experiment both CFU assay and microscopic observation indicated that the two biotypes were present in the ratio of approximately 1∶1 (Fig. 4). After 24 hours of coculturing CFU of O395 was below the detectable limit, however this decline was not corroborated microscopically as little change was observed in the relative ratio of O395 (green) and N16961 (red) (Fig. 4). These results suggested that although CFU of the classical biotype decreased drastically when cocultured with the El Tor biotype, lysis of the cells did not occur.

### Flow Cytometric Analysis

**Figure 5:**
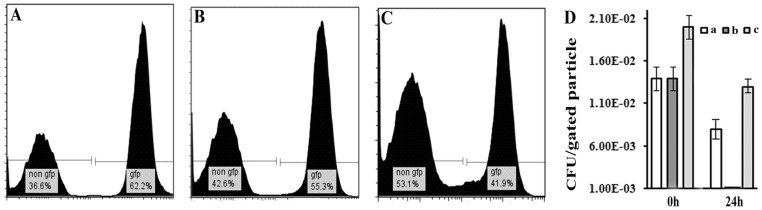
Flow cytometric analysis of cocultures. N16961 and GFP labeled O395 were grown separately for 24 hours, mixed (1∶1) and samples were removed from the cocultures for flow cytometric analyses at 24 hours (A), 48 hours (B) and 7 days (C) after mixing. D. The GFP labeled (b) and non- labeled cells (c) from the cocultures were separated by FACS at the time of mixing (0 h) and 24 hour after mixing, and CFU of each population was determined. O395 monocultures (a) were used as an experimental control. Plating efficiency was scored as CFU per particle sorted from each gated population. Data is represented as means ± SD, n = 3.

In view of the fact that the classical biotype cells are rendered non-culturable but still persist in cocultures with the El Tor biotype, the possibility that the classical biotype maybe converted to the viable non-culturable state (VBNC) in the cocultures was considered. For this purpose, flow cytometric analysis was performed with El Tor N16961 and GFP-labeled classical O395 (O395/pEGFP, Table S1). In a preliminary experiment, individual cultures of GFP-labeled O395 were grown for upto 7 days and aliquots were removed at regular intervals for CFU assay and flow cytometric analysis. The results obtained indicated that although part of the GFP label was lost by the O395 cells progressively with time, majority of the cells still retained GFP after 7 days (Fig. S4). Next, El Tor N16961 and GFP-labeled O395 were grown separately for 24 hours, mixed and samples were removed every 24 hours for 7 days for CFU assay and flow cytometric analysis. At the start of the coculture, CFU assay indicated that the two strains were in the ratio of approximately 1∶1 and flow cytometric analysis also indicated that GFP positive cells constituted about 60% of the population (Fig. 5A). After 24 hours, CFU of O395 in the cocultures decreased drastically to below detectable limits, yet flow cytometric analysis indicated little change in the GFP positive cell population (Fig. 5B). Even after 7 days, allowing for the loss of GFP label observed even in the individual cultures of O395-GFP (Fig. S4), the GFP-labeled population in cocultures remained almost unchanged (Fig. 5C).

To directly demonstrate that O395 in the cocultures was no longer culturable, GFP labeled O395 was separated from N16961 in the cocultures by FACS (>93% purity, Fig. S5) and the sorted O395 cells from the cocultures were plated on LB-agar or inoculated directly into LB broth. No growth of O395 was observed under either of the two conditions (Fig. 5D). That the loss of culturability was not due to the sorting process was demonstrated by CFU assay of individual O395 cultures sorted in an identical manner, which showed that culturability is retained in the individual cultures after sorting (Fig. 5D).

**Figure 6:**
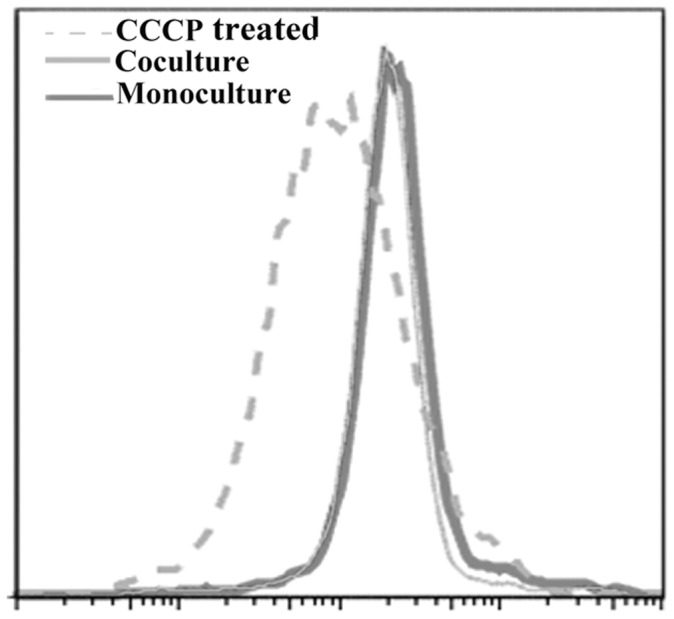
Viability assay of classical O395. O395 cells from mono- and co- cultures were sorted and stained with the dye JC-1 and analyzed by flow cytometry. Cells treated with CCCP for membrane depolarization was used as negative controls.

Taken together the results described so far clearly indicated that the classical biotype cells lost culturablity when cocultured with the El Tor biotype. Whether the non-culturable classical cells in the cocultures remained viable was next examined. For this purpose, O395 cells sorted from cocultures as well as from individual O395 cultures were treated with the membrane potential (Δφ)–sensitive dye JC-1 (5,5′,6,6′-tetrachloro-1,1′,3,3′-tetraethylbenzimi-dazolylcarbocyanine iodide) (Sigma), a sensitive, viability probe that can be used with flow cytometry [31]. FACS analysis of JC-1 stained cells indicated that there was practically no difference in membrane potential of the O395 cells sorted from cocultures and from individual cultures (Fig. 6) although CFU assay indicated that there was a drastic difference in culturability of the cells from these two cultures (Fig. 5D). When the sorted cell populations were treated with the protonophore CCCP (Carbonyl cyanide m-chlorophenyl hydrazone) to dissipate the cellular proton gradient, membrane depolarization of the cells was observed (Fig. 6). These results indicated that viability of the culturable O395 cells in individual cultures and the non-culturable O395 cells in cocultures was similar.

### DNase 1 Protection Assay

Another approach that was taken to further confirm that membrane integrity was maintained in the non-culturable classical cells in cocultures, was the DNase protection assay. Intact membranes protect genomic DNA from digestion by exogenous nucleases, whereas leaky or ruptured membranes allow entry of the nucleases and subsequent degradation of genomic DNA [32]. 24 hour cocultures of classical O395 and El Tor N16961 were treated with DNase1 for 3 hours and quantitative PCR was performed using O395 specific primers (*rstR*, Table S2). qPCR was also performed on samples without DNase treatment. In addition, control experiments were performed where cultures were heat killed to disrupt bacterial membranes before DNase treatment thus allowing the exogenously added DNase access to the bacterial DNA. A very significant increase in Ct values was obtained when the heat killed cells were exposed to DNase indicating that, as expected, these cells had lost membrane integrity (Table 1). However, O395 cells in cocultures, showed only a modest increase in Ct values when exposed to exogenous DNase (Table 1) suggesting that although these cells were non-culturable, they maintained membrane integrity and were indeed viable.

**Table 1:** DNase 1 protection assay of non-culturable cells of O395 and O395?*rpos* in cocultures.

Sample	Average Ct (± SD)	
	O395 in cocultures	O395Δ*rpos* in cocultures
Cells in buffer	21.18±0.21	20.01±0.54
Cells+DNase	23.52±1.14	23.24±1.11
Heat killed cells	21.08±0.25	19.96±0.25
Heat killed cells+DNase	30.84±1.26	29.83±0.43

### The Genetic Basis of Conversion of the Classical Biotype to the Non-culturable State in Cocultures with the El Tor Biotype

**Figure 7:**
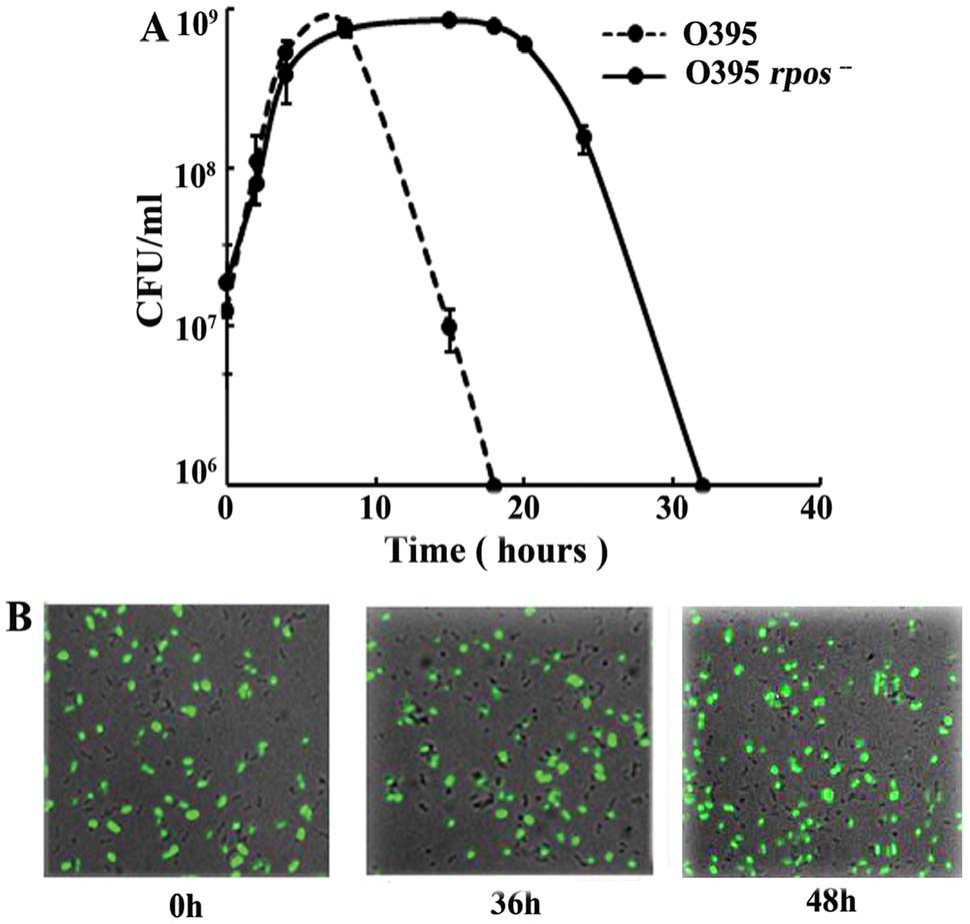
Delayed loss of culturability of *O395*Δ*rpos* in cocultures with El Tor N16961. A. Growth of O395?*rpos* and O395 in cocultures with El Tor N16961. Data is represented as means ± SD, n = 3. B. O395Δ*rpos* and El Tor N16961 were grown separately for 24 hours and mixed in the ratio of about 1∶1. Samples were removed at the time of mixing (0 h), 36 hours and 48 hours after mixing and processed for confocal microscopy.

To identify genetic factor(s) in the classical biotype responsible for its conversion to the non-culturable state, we presumed that if mutations occurred in these factors, the classical cells would retain culturability in the cocultures. Accordingly, a transposon-mutant library of strain O395 was mixed with El Tor N16961 and cocultured for 24 hours and O395 Tn mutants that survived in the cocultures were selected. A mutant was finally identified that remained culturable in the cocultures for a significantly longer time than the parent O395 cells (Fig. 7A). The mutant was shown to contain a Tn insertion in the *rpoS* gene, encoding the stationary phase specific sigma factor (supplementary information). When *O395Δrpos* and N16961 were cocultured it was observed that the *O395Δrpos* remained culturable until about 30 hours after which culturability decreased and by 48 hours *O395Δrpos* could not be detected in the cocultures by CFU assay (Fig. 7A). Microscopic observations indicated that the relative proportion of GFP labeled O395Δ*rpos* and N16961 remained practically unchanged even 48 hours after coculturing (Fig. 7B) indicating that the O395Δ*rpos* cells in coculture did not undergo lysis even after loss of culturability. Furthermore, DNase 1 protection assay clearly indicated that the genomic DNA was protected from exogenous DNase in the O395Δ*rpos* even after 48 hours of coculturing (Table 1), suggesting that membrane integrity of the cells was not compromised, hence the cells though non-culturable were viable. Taken together, these results indicate that although the *O395Δrpos* remained culturable for a longer period of time than the parent strain in cocultures with the El Tor biotype, the *O395Δrpos* strain finally lost culturability and was converted to the VBNC state.

Next to examine if RpoS has any role in the growth inhibitory activity of the El Tor strains, N16961*Δrpos* strain was cocultured with strain O395 or O395*Δrpos*. The results obtained with N16961*Δrpos* were similar to that obtained with strain N16961 indicating that the El Tor RpoS may not have a role in the VBNC conversion of the classical strain in cocultures (Fig. S6).

## Discussion

The El Tor biotype of *V. cholerae* is a highly adapted organism that within a decade displaced the classical biotype as the predominant cause of epidemic cholera [3]. To the best of our knowledge, such a rapid global elimination of a long established epidemic strain by a newly emerged closely related strain has not been observed for any other bacterial pathogen. The molecular and ecological events that led to this change in the epidemiology of cholera are still unknown.

When *V. cholerae* classical biotype was cultured with the El Tor biotype, a precipitous decline in the CFU of the classical biotype was observed (Fig. 1). The fact that this phenomenon occurred in the death phase and under alkaline conditions (Fig. 2) was reminiscent of the GASP phenotype, where coculturing wild type *E. coli* with a specific *rpoS* mutant (*rpoS* 819) resulted in a drastic decline in the wild type population [19], [20]. However, the specific *rpoS* mutation that confers the ability on *E. coli rpoS 819* mutant to eliminate the wild type cells is not present in the El Tor biotype and sequence of the *rpoS* gene is identical in the two biotypes, although RpoS might have a role in the loss of CFU of the classical biotype in cocultures with the El Tor biotype (Fig. 7).

One of the prerequisites for the decline of CFU of classical cells in the cocultures was physical contact with the El Tor cells (Fig. S2). In this respect, the phenomenon resembles the contact dependent inhibition (CDI) reported for *E. coli* but is distinct from CDI in several aspects. CDI occurs in the logarithmic phase of growth and is dependent on the *cdiA* and *cdiB* gene products [22]. In contrast, inhibition of the classical biotype occurred in the late stationary to death phase and the *cdiA* and *cdiB* genes or closely related homologs are not present in *V. cholerae*. More recently, stationary phase contact-dependent inhibition (SCDI) has been reported in *E. coli* [21]. In some ways, SCDI is similar to the inhibition of the classical biotype cells in cocultures with the El Tor biotype. Both processes occur in the stationary phase under alkaline pH conditions and require physical contact between the inhibitor and the target cells. On the other hand, there is one fundamental difference, SCDI has been attributed to mutations in the *glgC* gene encoding ADP-glucose pyrophosphorylase, an enzyme of the glycogen synthesis pathway and much higher levels of glycogen has been reported in the inhibitor as compared to the target cells [21]. However, no difference in glycogen accumulation was observed between classical O395 and El Tor N16961 (data not shown).

When *V. cholerae* classical biotype was cultured with the El Tor biotype, in spite of the drastic reduction in the CFU of the classical biotype observed (Fig. 1), several lines of evidence including microscopy (Fig. 4), flow cytometric analysis (Fig. 5) and measurement of DNA released into the supernatant (Fig. 3), indicate that there was practically no lysis of the classical cells for at least seven days after loss of culturability. It was intriguing to note that the classical biotype when cultured individually without the El Tor biotype underwent significant lysis in the death phase (Fig. 3). It is reasonable to hypothesize that coculturing the classical biotype with the El Tor biotype protects the former from lysis and allows the cells to remain viable in spite of the loss of culturability. Evidence for viability of the non-culturable classical cells in the cocultures with the El Tor biotype was obtained using primarily two approaches dependent on membrane integrity since retention of membrane integrity is a determining characteristic of viability.

It has been demonstrated that conversion of non-culturable *V. cholerae* to the culturable state occurs in rabbit ileal loops [33], human intestine [34] and when cocultured with eukaryotic cell lines [35]. We attempted to examine whether classical biotype cells converted to the VBNC state in cocultures with the El Tor biotype could be resuscitated to the culturable form in rabbit intestine or in the presence of the intestinal cell line INT407. For this purpose, the classical biotype cells after conversion to the VBNC state were separated from the El Tor biotype in the cocultures using flow cytometry and incubated with the INT407 cell line or inoculated into rabbit ileal loops. However 100% post sort purity of the classical biotype population could never be achieved (Fig. S5), and the El Tor cells present as a minority in the sorted classical population could not be removed by antibiotic treatment as non growing bacteria in the late stationary or death phase are recalcitrant to antibiotic treatment [36], [37]. These El Tor cells grew rapidly in rabbit intestines and in the INT 407 cocultures and might be expected to outcompete the classical biotype population even if there was a conversion of the VBNC classical cells to the culturable form, given the very slow rate reported for such conversions. Due to this technical limitation, it was not possible to arrive at any definite conclusion.

The viable non-culturable (VBNC) state of *V. cholerae* O1 and indeed the concept of VBNC was first reported by Xu *et al*. [38]. Subsequently, the existence of the VBNC state has been documented in at least 67 different bacterial species [25], [39]. The VBNC state is known to be induced by a number of physico-chemical stress conditions [39], [40], [41]. Induction of the VBNC state in a bacterial strain when cocultured with another (in this case, closely related) bacterial strain has, to the best of our knowledge not been reported previously and may have important implications in bacterial ecology and evolution. In Bangladesh, the El Tor biotype emerged in 1968 and by 1973 was thought to have completely replaced the classical biotype. Surprisingly in 1982, the classical biotype reappeared as the predominant epidemic strain in Bangladesh, only to disappear again in the late 1980s [42], [43]. It is attractive to hypothesize that upon advent of the El Tor biotype, the classical biotype may have been converted to the VBNC state in which it existed defying detection until some as yet unknown environmental or climatic condition favoured its resuscitation to the culturable state. Several such rounds of culturable to non-culturable conversions and vice-versa may account for the ‘mysterious disappearance and reappearance’ of the classical biotype in Bangladesh [43]. Indeed molecular analyses of strains isolated between 1961 and 1992 in Bangladesh support the contention that the classical biotype never completely disappeared from Bangladesh [44]. Furthermore, the VBNC classical biotype cells may form a reservoir of classical biotype genes that could be transferred to other serogroups or biotypes to account for the emergence of hybrid strains like the El Tor variants reported recently that have acquired certain classical biotype characteristics [45], [46]. Indeed the VBNC classical biotype may contribute to the changing epidemiology of global cholera in ways that need to be understood.

In conclusion, it has been demonstrated in this study that the competitive exclusion of the classical biotype by the El Tor biotype of *V. cholerae* could be reproduced in cocultures under standard laboratory conditions and the apparent inability to detect the classical biotype cells in the cocultures was due to their conversion to the non-culturable state although the cells remained viable. Further work is necessary to identify the genetic basis of this phenomenon.

## Supporting Information

Figure S1 The classical biotype strain O395 was cocultured with the El Tor biotype strains C6709 and E7946 and serogroup O139 strain SG-24. Bars represent the CFU of strain O395 in cocultures with the indicated El Tor strains at different time intervals. Values are given as means ± SD
(TIF)

Figure S2 The classical strain O395 and El Tor strain N16961 were grown separately to the late stationary phase (24 h) and the cultures were placed in the arms X and Y (separated by a 0.22 µm filter) of a modified U tube as follows. A: O395 in both arms X and Y; B: O395 in arm X and N16961 in arm Y; C: O395 and N16961 mixed cultures in both arms X and Y. The CFU of O395 was assayed in the samples A, B and C at the start of the experiment and after 24 hour incubation at 37°C.
(TIF)

Figure S3 Isolated genomic DNA (lane a) was incubated with 20 µl cell free supernatant of 24 hour grown cultures of strain N16961Δ*dns*Δ*xds* (lane b), strain N16961 (lane c) or strain O395 (lane d) and analyzed by agarose (1%) gel electrophoresis. No degradation of DNA by culture supernatants of strains N16961?*dns*?*xds* and O395 indicated absence of secreted DNase.
(TIF)

Figure S4 Flow cytometric analysis of GFP-labeled O395 grown individually in monocultures for 24 hours (A), 48 hours (B) and 7 days (C). The proportion of populations that have retained or lost the GFP label are indicated.
(TIF)

Figure S5 Purity of GFP-labeled O395 cells sorted by FACS from monocultures (A) and cocultures (B) 24 hours after mixing.
(TIF)

Figure S6 Strain N16961Δ*rpos* (Nal^r^) was cocultured with wild type strain O395 or O395Δ*rpos* and CFU of the strains was assayed at regular intervals. A: CFU of O395**/**CFU of N16961Δ*rpos*; B: CFU of O395Δ*rpos* **/**CFU of N16961Δ*rpos* in the cocultures.
(TIF)

Table S1 Bacterial strains and plasmids.
(DOC)

Table S2 Oligonucleotide primes.
(DOC)

Supporting Information S1 Materials and Methods.
(DOC)

## References

[1] Ryan ET (2011) Haiti in the context of the current global cholera pandemic. Emerg Infect Dis 17: 2175–2176. 22204041 10.3201/eid1711.110849 PMC3310588

[2] Kaper JB, Morris Jr JG, Levine MM (1995) Cholera. Clin Microbiol Rev 8: 48–86. 7704895 10.1128/cmr.8.1.48 PMC172849

[3] Faruque SM, Albert MJ, Mekalanos JJ (1998) Epidemiology, genetics, and ecology of toxigenic *Vibrio cholerae* . Microbiol Mol Biol Rev 62: 1301–1314. 9841673 10.1128/mmbr.62.4.1301-1314.1998 PMC98947

[4] Sack DA, Sack RB, Nair GB, Siddique AK (2004) Cholera. Lancet 363: 223–233. 14738797 10.1016/s0140-6736(03)15328-7

[5] Basu A, Garg P, Datta S, Chakraborty S, Bhattacharya T, et al (2000) *Vibrio cholerae* O139 in Calcutta, 1992–1998: incidence, antibiograms, and genotypes. Emerg Infect Dis 6: 139–147. 10756147 10.3201/eid0602.000206 PMC2640858

[6] Faruque SM, Sack DA, Sack RB, Colwell RR, Takeda Y, et al (2003) Emergence and evolution of *Vibrio cholerae* O139. Proc Natl Acad Sci U S A 100: 1304–1309. 12538850 10.1073/pnas.0337468100 PMC298768

[7] Yoon SS, Mekalanos JJ (2006) 2,3-butanediol synthesis and the emergence of the *Vibrio cholerae* El Tor biotype. Infect Immun 74: 6547–6556. 17015461 10.1128/IAI.00695-06 PMC1698044

[8] DiRita VJ, Neely M, Taylor RK, Bruss PM (1996) Differential expression of the ToxR regulon in classical and E1 Tor biotypes of *Vibrio cholerae* is due to biotype-specific control over *toxT* expression. Proc Natl Acad Sci U S A 93: 7991–7995. 8755590 10.1073/pnas.93.15.7991 PMC38862

[9] Murley YM, Behari J, Griffin R, Calderwood SB (2000) Classical and El Tor biotypes of *Vibrio cholerae* differ in timing of transcription of *tcpPH* during growth in inducing conditions. Infect Immun 68: 3010–3014. 10769005 10.1128/iai.68.5.3010-3014.2000 PMC97520

[10] Beyhan S, Tischler AD, Camilli A, Yildiz FH (2006) Differences in gene expression between the classical and El Tor biotypes of *Vibrio cholerae* O1. Infect Immun 74: 3633–3642. 16714595 10.1128/IAI.01750-05 PMC1479229

[11] Abuaita BH, Withey JH (2009) Bicarbonate Induces *Vibrio cholerae* virulence gene expression by enhancing ToxT activity. Infect Immun 77: 4111–4120. 19564378 10.1128/IAI.00409-09 PMC2738005

[12] Dziejman M, Balon E, Boyd D, Fraser CM, Heidelberg JF, et al (2002) Comparative genomic analysis of *Vibrio cholerae*: genes that correlate with cholera endemic and pandemic disease. Proc Natl Acad Sci U S A 99: 1556–1561. 11818571 10.1073/pnas.042667999 PMC122229

[13] Keasler SP, Hall RH (1993) Detecting and biotyping *Vibrio cholerae* O1 with multiplex polymerase chain reaction. Lancet 341: 1661. 8100020 10.1016/0140-6736(93)90792-f

[14] Chowdhury R, Biswas SK, Das J (1989) Abortive replication of choleraphage phi 149 in *Vibrio cholerae* biotype El Tor. J Virol 63: 392–397. 2908925 10.1128/jvi.63.1.392-397.1989 PMC247695

[15] Biswas SK, Chowdhury R, Das J (1992) A 14-kilodalton inner membrane protein of *Vibrio cholerae* biotype E1 Tor confers resistance to group IV choleraphage infection to classical vibrios. J Bacteriol 174: 6221–6229. 1400172 10.1128/jb.174.19.6221-6229.1992 PMC207691

[16] Takeya K, Otohuji T, Tokiwa H (1981) FK phage for differentiating the classical and El Tor groups of *Vibrio cholerae* . J Clin Microbiol 14: 222–224. 7276149 10.1128/jcm.14.2.222-224.1981 PMC271937

[17] Gonzalez-Pastor JE, Hobbs EC, Losick R (2003) Cannibalism by sporulating bacteria. Science 301: 510–513. 12817086 10.1126/science.1086462

[18] Steinmoen H, Knutsen E, Havarstein LS (2002) Induction of natural competence in *Streptococcus pneumoniae* triggers lysis and DNA release from a subfraction of the cell population. Proc Natl Acad Sci U S A 99: 7681–7686. 12032343 10.1073/pnas.112464599 PMC124321

[19] Zambrano MM, Siegele DA, Almiron M, Tormo A, Kolter R (1993) Microbial competition: *Escherichia coli* mutants that take over stationary phase cultures. Science 259: 1757–1760. 7681219 10.1126/science.7681219

[20] Farrell MJ, Finkel SE (2003) The growth advantage in stationary-phase phenotype conferred by *rpoS* mutations is dependent on the pH and nutrient environment. J Bacteriol 185: 7044–7052. 14645263 10.1128/JB.185.24.7044-7052.2003 PMC296246

[21] Lemonnier M, Levin BR, Romeo T, Garner K, Baquero MR, et al (2008) The evolution of contact-dependent inhibition in non-growing populations of *Escherichia coli* . Proc Biol Sci 275: 3–10. 17956846 10.1098/rspb.2007.1234 PMC2562405

[22] Aoki SK, Pamma R, Hernday AD, Bickham JE, Braaten BA, et al (2005) Contact-dependent inhibition of growth in *Escherichia coli* . Science 309: 1245–1248. 16109881 10.1126/science.1115109

[23] Pukatzki S, Ma AT, Sturtevant D, Krastins B, Sarracino D, et al (2006) Identification of a conserved bacterial protein secretion system in *Vibrio cholerae* using the *Dictyostelium* host model system. Proc Natl Acad Sci U S A 103: 1528–1533. 16432199 10.1073/pnas.0510322103 PMC1345711

[24] MacIntyre DL, Miyata ST, Kitaoka M, Pukatzki S (2010) The *Vibrio cholerae* type VI secretion system displays antimicrobial properties. Proc Natl Acad Sci U S A 107: 19520–19524. 20974937 10.1073/pnas.1012931107 PMC2984155

[25] Oliver JD (2005) The viable but nonculturable state in bacteria. J Microbiol 43 Spec No: 93 100. 15765062

[26] Pradhan S, Baidya AK, Ghosh A, Paul K, Chowdhury R (2010) The El Tor biotype of *Vibrio cholerae* exhibits a growth advantage in the stationary phase in mixed cultures with the classical biotype. J Bacteriol 192: 955–963. 20023022 10.1128/JB.01180-09 PMC2812972

[27] Rubin EJ, Akerley BJ, Novik VN, Lampe DJ, Husson RN, et al (1999) In vivo transposition of mariner-based elements in enteric bacteria and mycobacteria. Proc Natl Acad Sci U S A 96: 1645–1650. 9990078 10.1073/pnas.96.4.1645 PMC15546

[28] McFall E, Newman EB (1996) Amino acids as carbon sources; In Neidhardt FC, et al., editors. *Escherichia coli* and *Salmonella*: cellular and molecular biology. Washington, DC: ASM Press. pp. 358–379.

[29] Blokesch M (2008) Schoolnik GK (2008) The extracellular nuclease Dns and its role in natural transformation of *Vibrio cholerae* . J Bacteriol 190: 7232–7240. 18757542 10.1128/JB.00959-08 PMC2580679

[30] Kimsey HH, Waldor MK (1998) CTXphi immunity: application in the development of cholera vaccines. Proc Natl Acad Sci U S A 95: 7035–7039. 9618534 10.1073/pnas.95.12.7035 PMC22729

[31] Smiley ST, Reers M, Mottola-Hartshorn C, Lin M, Chen A, et al (1991) Intracellular heterogeneity in mitochondrial membrane potentials revealed by a J-aggregate-forming lipophilic cation JC-1. Proc Natl Acad Sci U S A 88: 3671–3675. 2023917 10.1073/pnas.88.9.3671 PMC51514

[32] Pawlowski DR, Metzger DJ, Raslawsky A, Howlett A, Siebert G, et al (2011) Entry of *Yersinia pestis* into the viable but nonculturable state in a low-temperature tap water microcosm. PLoS One 6: e17585. 21436885 10.1371/journal.pone.0017585 PMC3059211

[33] Colwell RR, Brayton PR, Grimes DJ, Roszak DR, Huq SA, et al (1985) Viable, but non-culturable *Vibrio cholerae* and related pathogens in the environment: implication for release of genetically engineered microorganisms. Bio/Technology 3: 817–820.

[34] Colwell RR, Brayton PR, Herrington D, Tall BD, Huq A, et al (1996) Viable but nonculturable *Vibrio cholerae* O1 revert to a culturable state in human intestine. World J Microb Biotechnol 12: 28–31. 10.1007/BF00327795 24415083

[35] Senoh M, Ghosh-Banerjee J, Ramamurthy T, Hamabata T, Kurakawa T, et al (2010) Conversion of viable but nonculturable *Vibrio cholerae* to the culturable state by co-culture with eukaryotic cells. Microbiol Immunol 54: 502–507. 20840148 10.1111/j.1348-0421.2010.00245.x

[36] Levin BR, Rozen DE (2006) Non-inherited antibiotic resistance. Nat Rev Microbiol 4: 556–562. 16778840 10.1038/nrmicro1445

[37] Nguyen D, Joshi-Datar A, Lepine F, Bauerle E, Olakanmi O, et al (2011) Active starvation responses mediate antibiotic tolerance in biofilms and nutrient-limited bacteria. Science 334: 982–986. 22096200 10.1126/science.1211037 PMC4046891

[38] Xu H-S, Roberts N, Singleton FL, Attwell RW, Grimes DJ, et al (1982) Survival and viability of nonculturable *Escherichia coli* and *Vibrio cholerae* in the estuarine and marine environment. Microb Ecol 8: 313–323. 24226049 10.1007/BF02010671

[39] Oliver JD (2010) Recent findings on the viable but nonculturable state in pathogenic bacteria. FEMS Microbiol Rev 34: 415–425. 20059548 10.1111/j.1574-6976.2009.00200.x

[40] Gunasekera TS, Sørensen A, Attfield PV, Sørensen SJ, Veal DA (2002) Inducible gene expression by nonculturable bacteria in milk after pasteurization. Appl Environ Microbiol 68: 1988–1993. 11916722 10.1128/AEM.68.4.1988-1993.2002 PMC123843

[41] Oliver JD, Dagher M, Linden K (2005) Induction of *Escherichia coli* and *Salmonella typhimurium* into the viable but nonculturable state following chlorination of wastewater. J Water Health 3: 249–257. 16209029 10.2166/wh.2005.040

[42] Samadi AR, Huq MI, Shahid N, Khan MU, Eusof A, et al (1983) Classical *Vibrio cholerae* biotype displaces El Tor in Bangladesh. Lancet 1: 805–807. 6132141 10.1016/s0140-6736(83)91860-3

[43] Siddique AK, Baqui AH, Eusof A, Haider K, Hossain MA, et al (1991) Survival of classic cholera in Bangladesh. Lancet 337: 1125–1127. 1674016 10.1016/0140-6736(91)92789-5

[44] Faruque SM, Alim AR Abdul, Rahman MM, Siddique AK, Sack RB, et al (1993) Clonal Relationships among Classical *Vibrio-Cholerae* 01 Strains Isolated between 1961 and 1992 in Bangladesh. J Clin Microbiol 31: 2513–2516. 7691878 10.1128/jcm.31.9.2513-2516.1993 PMC265789

[45] Udden SM, Zahid MS, Biswas K, Ahmad QS, Cravioto A, et al (2008) Acquisition of classical CTX prophage from *Vibrio cholerae* O141 by El Tor strains aided by lytic phages and chitin-induced competence. Proc Natl Acad Sci U S A 105: 11951–11956. 18689675 10.1073/pnas.0805560105 PMC2575248

[46] Nair GB, Faruque SM, Bhuiyan NA, Kamruzzaman M, Siddique AK, et al (2002) New variants of *Vibrio cholerae* O1 biotype El Tor with attributes of the classical biotype from hospitalized patients with acute diarrhea in Bangladesh. J Clin Microbiol 40: 3296–3299. 12202569 10.1128/JCM.40.9.3296-3299.2002 PMC130785

