# Supplementary figures and images for: Vibrio cholerae Classical Biotype Is Converted to the Viable Non-Culturable State when Cultured with the El Tor Biotype
Source: PLoS One. 2013 Jan 9;8(1):e53504. doi: 10.1371/journal.pone.0053504 (PMC3541145; doi:10.1371/journal.pone.0053504)

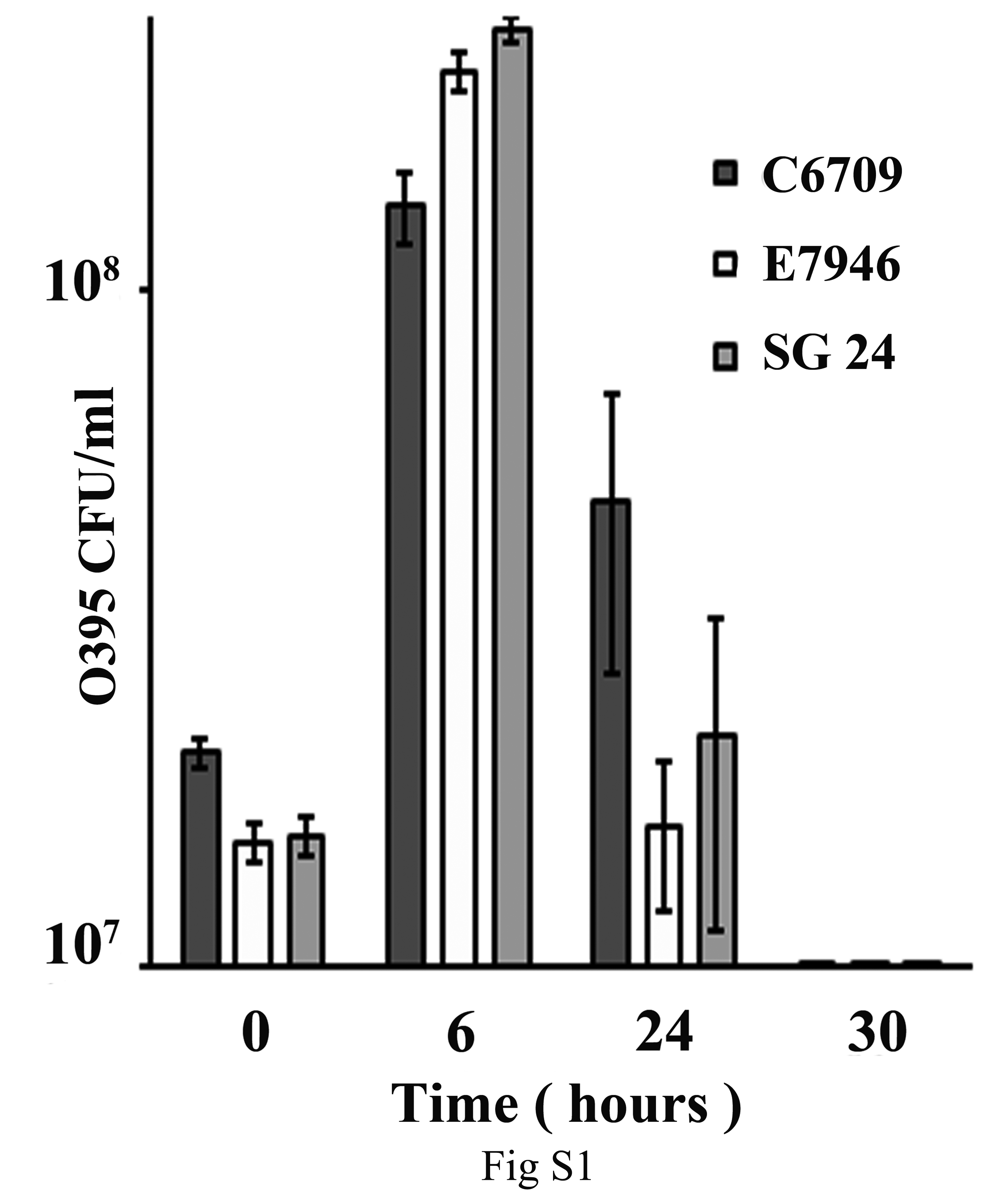

Supplement: Figure S1 — The classical biotype strain O395 was cocultured with the El Tor biotype strains C6709 and E7946 and serogroup O139 strain SG-24. Bars represent the CFU of strain O395 in cocultures with the indicated El Tor strains at different time intervals. Values are given as means ± SD (TIF) [file pone.0053504.s001.tif]

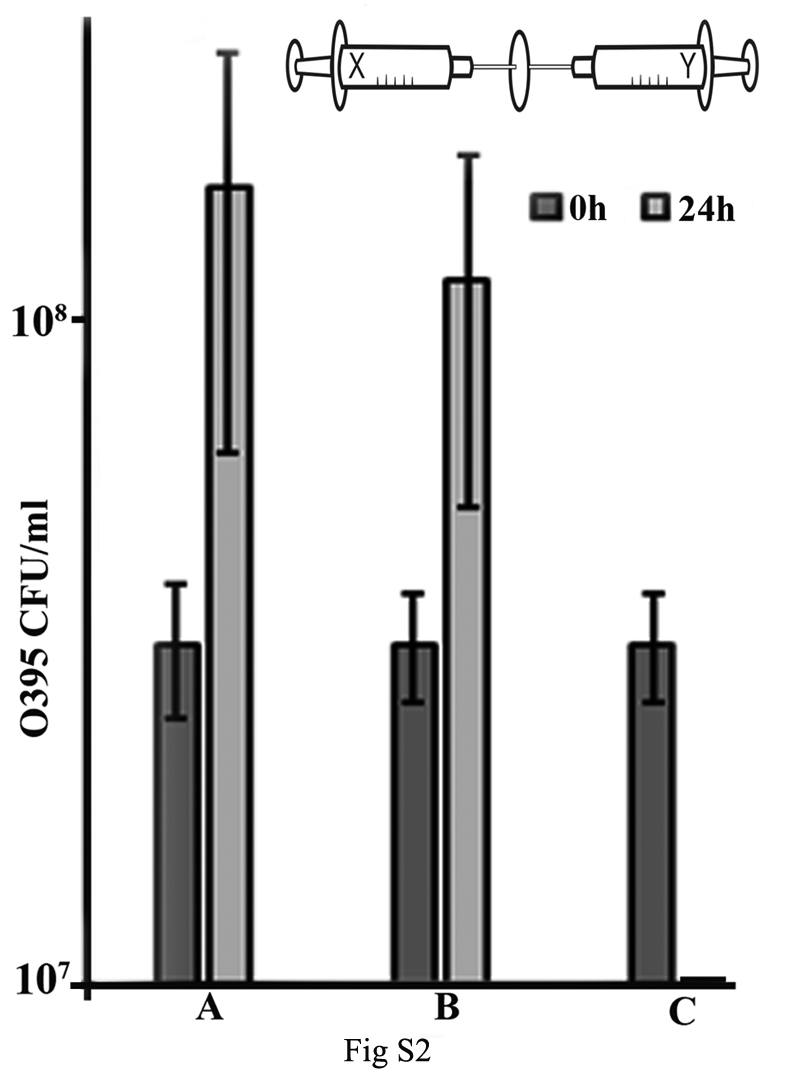

Supplement: Figure S2 — The classical strain O395 and El Tor strain N16961 were grown separately to the late stationary phase (24 h) and the cultures were placed in the arms X and Y (separated by a 0.22 µm filter) of a modified U tube as follows. A: O395 in both arms X and Y; B: O395 in arm X and N16961 in arm Y; C: O395 and N16961 mixed cultures in both arms X and Y. The CFU of O395 was assayed in the samples A, B and C at the start of the experiment and after 24 hour incubation at 37°C. (TIF) [file pone.0053504.s002.tif]

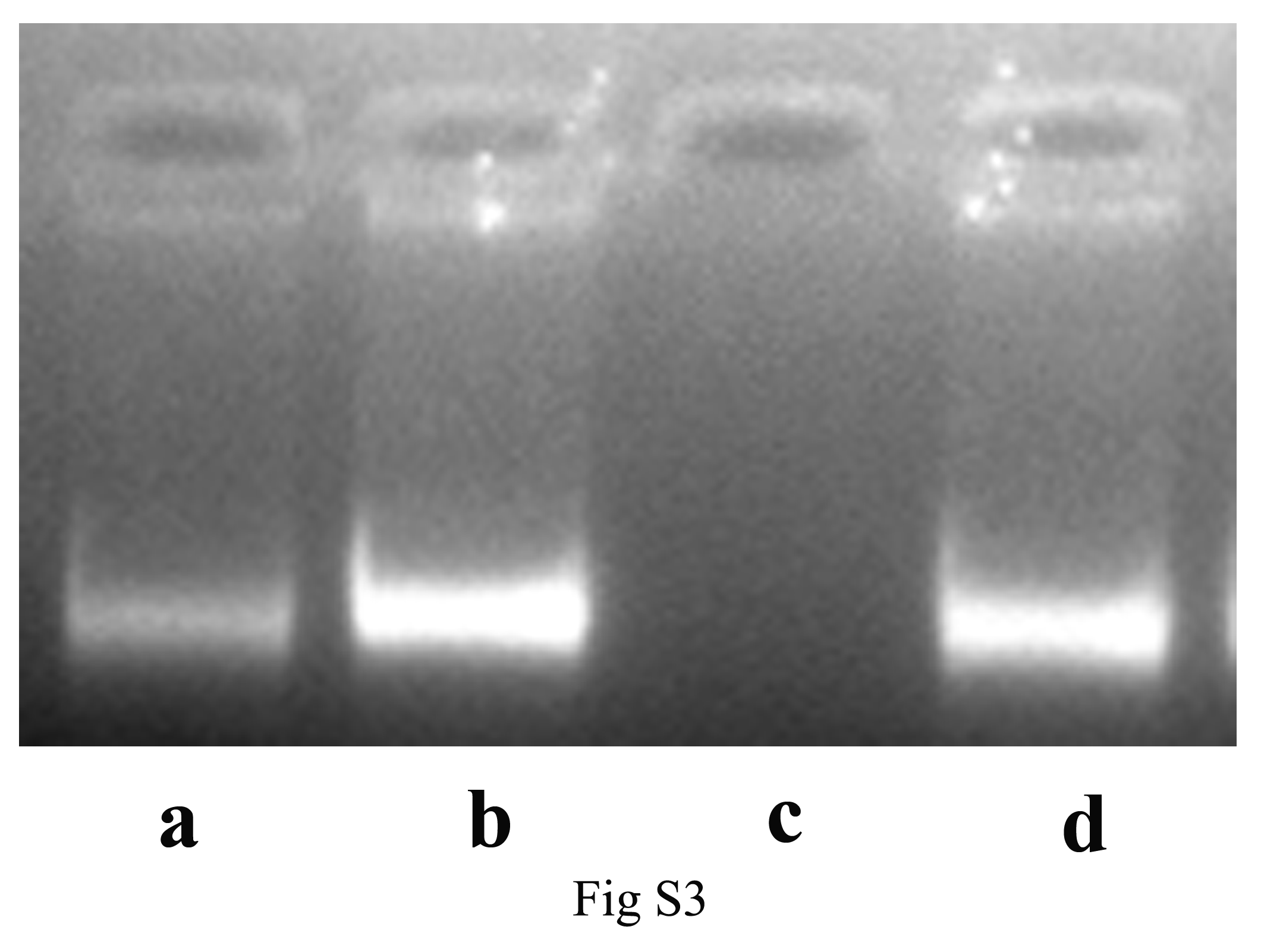

Supplement: Figure S3 — Isolated genomic DNA (lane a) was incubated with 20 µl cell free supernatant of 24 hour grown cultures of strain N16961ΔdnsΔxds (lane b), strain N16961 (lane c) or strain O395 (lane d) and analyzed by agarose (1%) gel electrophoresis. No degradation of DNA by culture supernatants of strains N16961?dns?xds and O395 indicated absence of secreted DNase. (TIF) [file pone.0053504.s003.tif]

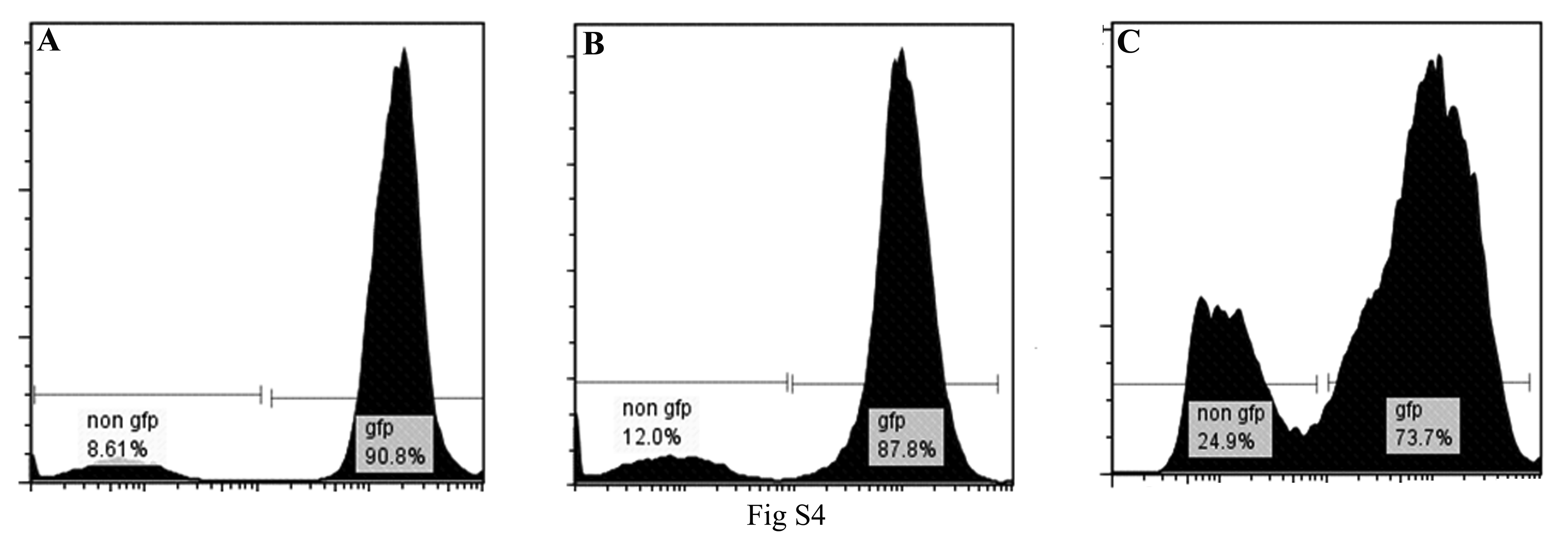

Supplement: Figure S4 — Flow cytometric analysis of GFP-labeled O395 grown individually in monocultures for 24 hours (A), 48 hours (B) and 7 days (C). The proportion of populations that have retained or lost the GFP label are indicated. (TIF) [file pone.0053504.s004.tif]

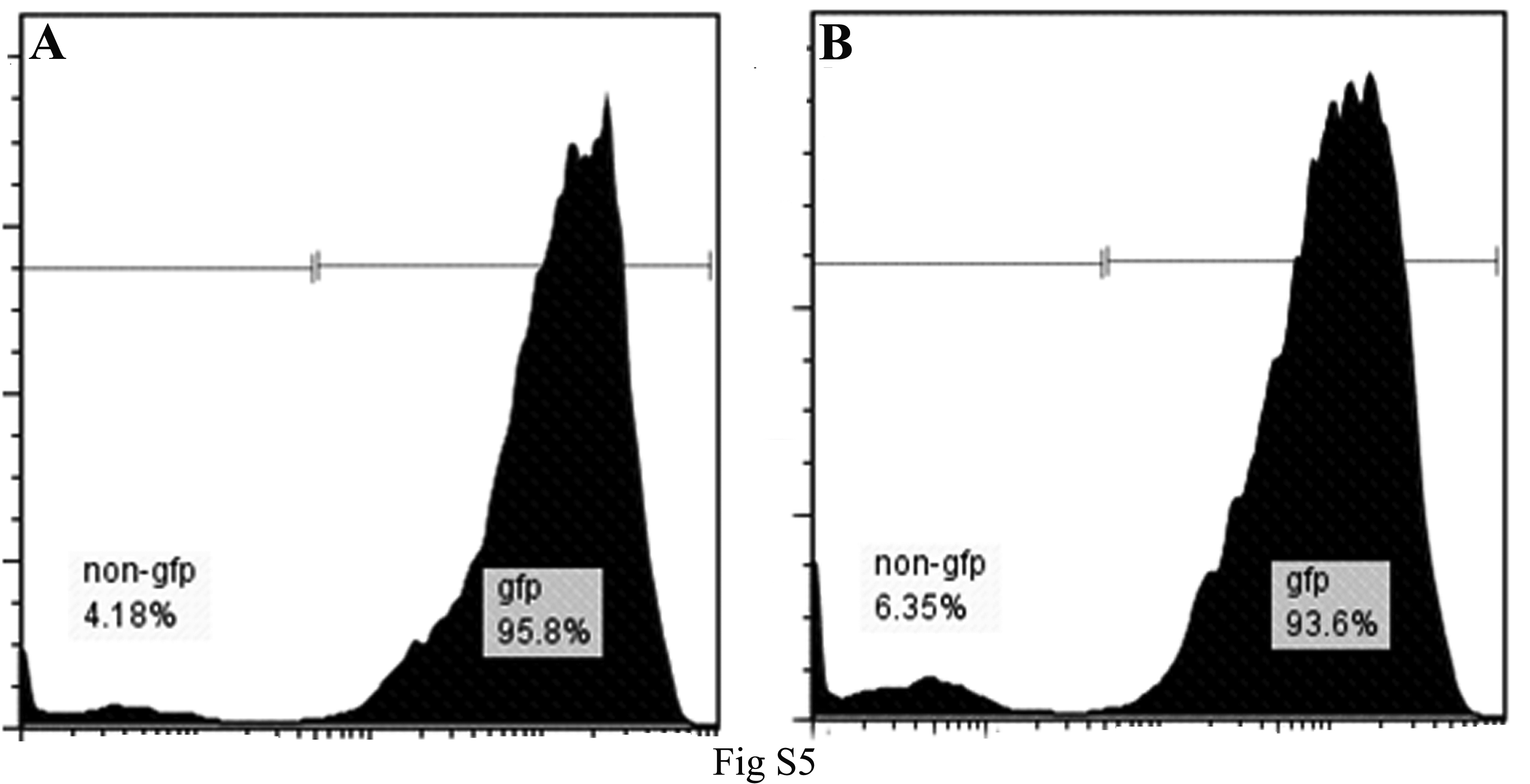

Supplement: Figure S5 — Purity of GFP-labeled O395 cells sorted by FACS from monocultures (A) and cocultures (B) 24 hours after mixing. (TIF) [file pone.0053504.s005.tif]

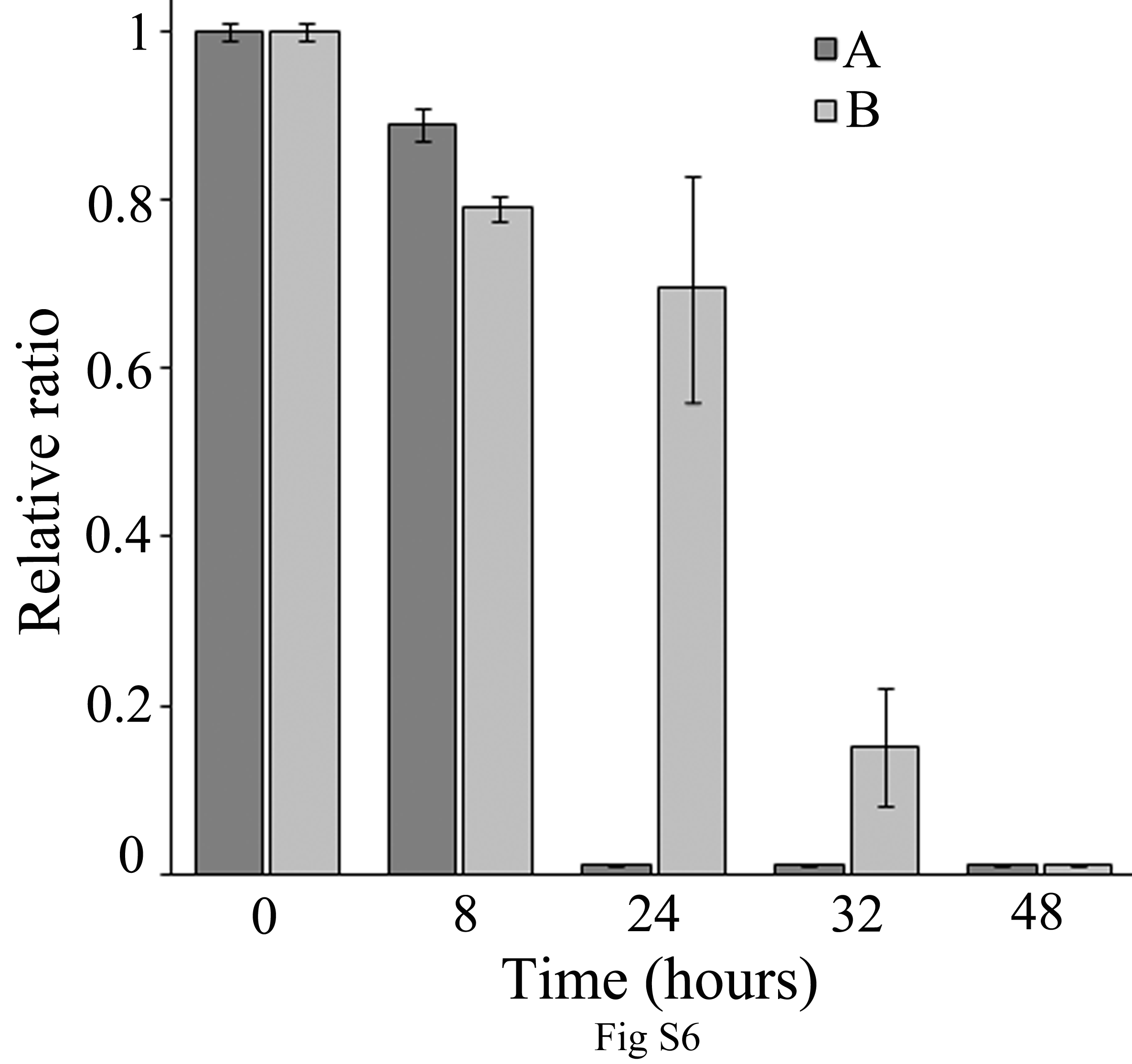

Supplement: Figure S6 — Strain N16961Δrpos (Nalr) was cocultured with wild type strain O395 or O395Δrpos and CFU of the strains was assayed at regular intervals. A: CFU of O395/CFU of N16961Δrpos; B: CFU of O395Δrpos /CFU of N16961Δrpos in the cocultures. (TIF) [file pone.0053504.s006.tif]
