# Supplementary material for: Vibrio cholerae Classical Biotype Is Converted to the Viable Non-Culturable State when Cultured with the El Tor Biotype
Source: PLoS One. 2013 Jan 9;8(1):e53504. doi: 10.1371/journal.pone.0053504 (PMC3541145; doi:10.1371/journal.pone.0053504)
Supplement: Table S1 — Bacterial strains and plasmids. (DOC) [file pone.0053504.s007.doc]

**Table S1.** Bacterial strains and plasmids used in this study

| **Strain / plasmid** | **Relevant characteristics** | **Source/Reference** |
| --- | --- | --- |
| O395 Sm^r^ | O1 classical; derivative of wild- type O395 | JJ Mekalanos, Harvard Medical School, Boston |
| O395 Sm^r^ Nal^r^ | O1 classical; derivative of O395 Sm^r^ | Laboratory collection |
| 569B | O1 classical; wild type | Laboratory collection |
| N16961 Sm^r^ | Ol El Tor; derivative of wild- type N16961 | Laboratory collection |
| N16961 Sm^r^ Nal^r^ | Ol El Tor; derivative of N16961 Sm^r^ | Laboratory collection |
| O395 *∆rpoS* Sm^r^  Kan^r^ | Transposon insertion in the *rpo*s gene | This study |
| N16961 *∆rpoS* Sm^r^ Nal^r^ Ap^r^ | N16961 *rpoS*::pGP704 with a disruption in the *rpos* gene | Laboratory collection |
| N16961*∆dns* | *∆dns* derivative of N16961 | 1 |
| N16961*∆dns∆xds* Sm^r^ Ap^r^ | N16961∆*dns xds*::pGP704 mutant incapable of producing extracellular DNase | This study |
| C6709 Sm^r^ | O1 El Tor | NICED, Kolkata, India |
| E7946 | O1 El Tor | NICED, Kolkata, India |
| SG-24 Sm^r^ | Serogroup O139 | NICED, Kolkata, India |
| pGP704 | *oriR6K mobRP4*, Ap^r^ |  |
| pFD1 | pGP704 carrying *Himar 1* based transposon; Km^r^ |  |
| pEGFP | Green fluorescent protein under control of *lac* promoter, Ap^r^ | S. Raychaudhuri, IMTECH, Chandigarh, India |
| pDsRed | Red fluorescent protein under control of *lac* promoter, Ap^r^ |  |

1. Blokesch M, Schoolnik GK (2008) The extracellular nuclease Dns and its role in natural transformation of *Vibrio cholerae*. J Bacteriol 190:7232-7240.
2. Miller VL, Mekalanos JJ (1988) A novel suicide vector and its use in the construction of insertion mutations: osmoregulation of outer membrane proteins and virulence determinants in *Vibrio cholerae* requires *toxR*. J Bacteriol 170: 2575-2583.
3. Rubin EJ, Akerley BJ, Novik VN, Lampe DJ, Husson RN, et al. (1999) In vivo transposition of mariner-based elements in enteric bacteria and mycobacteria. Proc Natl Acad Sci U S A 96:1645-1650.
4. Fernandes PJ, Guo Q, Donnenberg MS (2007) Functional consequences of sequence variation in bundlin, the enteropathogenic *Escherichia coli* type IV pilin protein. Infect Immun 75:4687-4696.
