# Supplementary material for: Vibrio cholerae Classical Biotype Is Converted to the Viable Non-Culturable State when Cultured with the El Tor Biotype
Source: PLoS One. 2013 Jan 9;8(1):e53504. doi: 10.1371/journal.pone.0053504 (PMC3541145; doi:10.1371/journal.pone.0053504)
Supplement: Table S2 — Oligonucleotide primes. (DOC) [file pone.0053504.s008.doc]

**Table S2: Oligonucleotide primes used in this study**

| Primers | Sequence (5′→3′) |
| --- | --- |
| rstR (F) | TCACCAAAACTGGAAACTGT |
| rstR (R) | ATTCTTTTTGTATTTCTCGACT |
| Mariner 1 | GGCCACGCGTGCACTAGTACNNNNNNNNNNTACNG |
| Mariner 2 | ATGCATTTAATACTAGCGACGC |
| Mariner 3 | GGCCACGCGTGCACTAGTAC |
| Mariner 4 | GCCATCTATGTGTCAGACCGG |
| xds 1 | TCAGCACCAGCATAGGATC |
| xds 2 | CCCCAACCTTTAAAGTCAA |
