# Supplementary material for: Vibrio cholerae Classical Biotype Is Converted to the Viable Non-Culturable State when Cultured with the El Tor Biotype
Source: PLoS One. 2013 Jan 9;8(1):e53504. doi: 10.1371/journal.pone.0053504 (PMC3541145; doi:10.1371/journal.pone.0053504)
Supplement: Supporting Information S1 — Materials and Methods. (DOC) [file pone.0053504.s009.doc]

# Supplementary information

**SI Materials and Methods**

**Construction of N16961∆*dns*∆*xds* mutant**

The N16961∆*dns* strain was provided by G Schoolnik, Stanford University . An insertion mutation in the *xds* gene (encoding a second extracellular DNase) of strain N16961∆*dns* was constructed. An internal fragment of the *xds* gene was PCR amplified using primers xds1 and xds2 (Table S2), cloned in the suicide vector pGP704 (Ap^r^) , transferred to N16961∆*dns*  (Sm^r^) and Sm^r^ Ap^r^ exconjugates were selected in which the suicide plasmid had integrated into the *V. cholerae* chromosome by a single crossover in the *xds* gene region.

**Quantitation of DNA in culture supernatants**

The classical O395 and El Tor N16961 Δ*xds* Δ*dns* strains were grown separately for 24 hours and appropriate amount of the El Tor biotype cell pellet was added to classical biotype culture so as to maintain the initial cell number of the two biotypes in the ratio of approximately 1:1. At regular intervals, samples were removed for CFU assay and isolation of DNA from culture supernatants. Cell free culture supernatants were prepared by centrifugation (6500 rpm, 10 min) followed by filtration through 0. 22 µm syringe filters. 100 µl of the filtrate was plated to ensure complete removal of bacteria. DNA was extracted from the filtrates, diluted 20 times and 2 µl was used as template for quantitative real time PCR using a SYBR green qPCR kit (Finnzyme) following the manufacturer’s protocol.

**Flow cytometric analysis**

Flow cytometric analysis and sorting were performed using a BD Influx stream in air sorter with a 70 μm sorting nozzle and sheath and sample pressure set at 10 and 10.8 psi respectively. GFP was excited using a 488 nm blue laser and signal was recorded through a 530/30 band pass filter. Spigot software was used for data acquisition and a threshold was applied on the SSC parameter. FlowJo software was used to analyze the stored data and percent GFP positive cells was obtained by gating. For sorting, the instrument was set up in a purity-yield mode. Two way sorting was performed with a rate of 8000 events/second and approximately 3000000 sorted particles were collected for each gated population in 5 ml polypropylene tubes. Post sort, an aliquot of the sorted cells was checked for purity.

**Tn mutagenesis of strain O395 and screening of mutant pool**

A transposon mutant library was constructed by transfer of the suicide plasmid pFD1 carrying a *Himar* 1 based minitransposon (Km^r^) from *E. coli* Sm10 λpir to *V. cholerae* O395 (Sm^r^) by conjugation. The donor and recipient strains were coincubated on LB agar for 6 hours at 37°C, transferred to LB containing streptomycin  (Sm 100 μg/ml) and IPTG (1 mM) and incubated at 37°C for 16 hours. The pool of *V. cholerae* O395 containing transposon insertions was selected using Sm (100 μg/ml) and Km (200 μg/ml). The O395 mutant pool was cocultured with the El Tor strain N16961 for 24 hours and O395 mutants (Sm^r^ Km^r^) that survived in the cocultures was selected.

**Identification of site of Tn insertion**

To determine the location of the transposon in the O395 mutant genome a first round of PCR was carried out with the primers Mariner1 and Mariner 2 followed by a round of nested PCR with the primers Mariner 3 and Mariner 4 (Table S2). The program for the two PCRs are as follows. First round PCR: 95°C 8 min; 6 × : 95°C 30 sec, 30°C 2 min, 72°C 1.5 min + 5 second increase per cycle; 30 × : 95°C 30 sec, 45°C 1 min, 72°C 2 min + 5 second increase per cycle; 72°C 7 min, 4°C hold. Second round PCR: 95°C 8 min; 35 × : 95°C 45 sec, 55°C 45 sec, 72°C 1.5 min +5 sec increase per cycle: 72°C 7 min, 4°C hold. The PCR product was purified and sequenced in a ABI 3130 xl Genetic Analyzer using the primer Mariner 4 (Table S2). The sequence obtained was matched with the *V. cholerae* genome data base using the BLAST algorithm.

**References**

1. Blokesch M, Schoolnik GK (2008) The extracellular nuclease Dns and its role in natural transformation of *Vibrio cholerae*. J Bacteriol 190: 7232-7240.

2. Miller VL, Mekalanos JJ (1988) A novel suicide vector and its use in the construction of insertion mutations: osmoregulation of outer membrane proteins and virulence determinants in *Vibrio cholerae* requires *toxR*. J Bacteriol 170: 2575-2583.

3. Rubin EJ, Akerley BJ, Novik VN, Lampe DJ, Husson RN, et al. (1999) In vivo transposition of mariner-based elements in enteric bacteria and mycobacteria. Proc Natl Acad Sci U S A 96: 1645-1650.
